# Development of a Field Guide for Assessing Readiness to Implement Evidence-Based Cancer Screening Interventions in Primary Care Clinics

**DOI:** 10.5888/pcd19.210395

**Published:** 2022-05-12

**Authors:** Sarah D. Hohl, Stephanie Melillo, Thuy T. Vu, Cam Escoffery, Amy DeGroff, Dara Schlueter, Leslie W. Ross, Annette E. Maxwell, Krishna P. Sharma, Jennifer Boehm, Djenaba Joseph, Peggy A. Hannon

**Affiliations:** 1 Health Promotion Research Center, University of Washington, Seattle, Washington; 2 Division of Cancer Prevention and Control, National Center for Chronic Disease Prevention and Health Promotion, Centers for Disease Control and Prevention, Atlanta, Georgia; 3 Rollins School of Public Health, Emory University, Atlanta, Georgia; 4 Fielding School of Public Health, University of California, Los Angeles, California

## Abstract

Evidence-based interventions, including provider assessment and feedback, provider reminders, patient reminders, and reduction of structural barriers, improve colorectal cancer screening rates. Assessing primary care clinics’ readiness to implement these interventions can help clinics use strengths, identify barriers, and plan for success. However, clinics may lack tools to assess readiness and use findings to plan for successful implementation. To address this need, we developed the Field Guide for Assessing Readiness to Implement Evidence-Based Cancer Screening Interventions (Field Guide) for the Centers for Disease Control and Prevention’s (CDC’s) Colorectal Cancer Control Program (CRCCP). We conducted a literature review of evidence and existing tools to measure implementation readiness, reviewed readiness tools from selected CRCCP award recipients (n = 35), and conducted semi-structured interviews with key informants (n = 8). We sought feedback from CDC staff and recipients to inform the final document. The Field Guide, which is publicly available online, outlines 4 assessment phases: 1) convene team members and determine assessment activities, 2) design and administer the readiness assessment, 3) evaluate assessment data, and 4) develop an implementation plan. Assessment activities and tools are included to facilitate completion of each phase. The Field Guide integrates implementation science and practical experience into a relevant tool to bolster clinic capacity for implementation, increase potential for intervention sustainability, and improve colorectal cancer screening rates, with a focus on patients served in safety net clinic settings. Although this tool was developed for use in primary care clinics for cancer screening, the Field Guide may have broader application for clinics and their partners for other chronic diseases.

**Summary**

**What is already known on this topic?**

Assessing readiness to implement evidence-based interventions that increase colorectal cancer screening can help organizations use strengths, identify barriers, and plan for success. However, primary care clinics lack tools to systematically assess readiness and develop relevant implementation plans.

**What is added by this report?**

We describe a field guide to assist organizations in collecting, evaluating, and using assessment data to develop practical plans that enhance implementation of cancer screening interventions.

**What are the implications for public health practice?**

The field guide integrates implementation science and practical experience into a tool to help clinics select and implement effective interventions to increase cancer screening. This tool can be tailored to address other chronic disease interventions.

## Background

In 2018 in the US, 141,074 men and women were diagnosed with colorectal cancer (CRC), the second leading cause of cancer death among cancers that affect both men and women (1). The US Preventive Services Task Force recommends screening for adults aged 45–75 (2). Despite the known benefits of screening for reducing CRC incidence and mortality (3–5), considerable work remains to reach the National Colorectal Cancer Roundtable goal of 80% of eligible individuals screened in every community (6) and the Healthy People 2030 goal of 74.4% of patients up to date with CRC screening (7). Only 65.2% of eligible individuals in the US, and fewer than half of eligible patients at federally qualified health centers (FQHCs), were up to date with CRC screening in 2018 (7,8). Moreover, people served by FQHCs, including Hispanic, African American, and American Indian/Alaska Native populations; those living in rural areas or below the federal poverty level; those without health insurance; and those with less than a high school education have poor CRC outcomes, due in part to lower screening rates (9–11).

Implementing interventions that combine 2 or more evidence-based approaches recommended by the Community Preventive Services Task Force — such as patient/client reminders, provider reminders, and provider assessment and feedback — increases CRC screening rates (12). Integrating these approaches into clinical care requires support for health systems and clinic-level change (13). To facilitate that support, increase screening rates, and reduce disparities, the Centers for Disease Control and Prevention (CDC) initiated the Colorectal Cancer Control Program (CRCCP) in 2009 (14). This was the first of 3 sequential 5-year cooperative agreements for the program, which funds state health departments, tribal organizations, universities, and other organizations (recipients) to partner with primary care clinics to increase CRC screening rates by supporting health system change. During the most recently completed funding cycle (2015–2020), recipients partnered with more than 800 primary care clinics, most of which were part of FQHCs, to implement 4 evidence-based interventions (EBIs) recommended by the Community Preventive Services Task Force (15) (Table 1).

**Table 1:** Evidence-Based Interventions Prioritized by the Centers for Disease Control and Prevention’s Colorectal Cancer Control Program

Evidence-based intervention	Definition
Patient or client reminders	These reminders include written messages (ie, letter, postcard, email, or text message) or telephone messages (including recorded or automated messages) advising patients that they are due for screening. Patient reminders can be general to reach a group of people or tailored to reach 1 person.
Provider reminders	Reminders inform health care providers that a patient is due or past due for a cancer screening test. A recall is another form of provider reminder that alerts providers that a client is overdue for screening. The reminders can be provided in different ways, such as in patient charts or by e-mail.
Provider assessment and feedback	Interventions that evaluate provider performance in delivering or offering screening to patients are called *assessments*. Presentation of information to providers about their performance in providing screening services is called *feedback*.
Reducing structural barriers	Structural barriers are noneconomic burdens or obstacles — such as inconvenient clinic hours or lack of transportation — that make it difficult for people to access cancer screening.

Implementation readiness — an organization’s combined capacity, commitment, and willingness to implement a new program, policy, or practice — facilitates implementation success (16–18). Because public health resources are limited, identifying a clinic’s readiness to successfully implement and sustain interventions, as well as gaps in clinic resources or practices that need to be addressed before implementation, is critical. Such assessment practices can guide clinics to select interventions with the greatest potential for long-term sustainability, and in turn help maximize the impact of public health spending, optimize clinic success, reduce cancer disparities, and improve population health.

The field of implementation science has introduced multiple theories, methods, and frameworks to assess implementation readiness and to help organizations consider and prepare for changing demands, priorities, and contexts (19). For example, more than 50 measurement tools described in 3 seminal systematic reviews assess some dimension of organizational implementation readiness (20–22). However, to our knowledge no existing tools apply specifically to readiness to implement cancer screening interventions, and only 1 has been developed for use specifically in primary care settings (23). Moreover, these resources may lack guidance to help organizations use assessment findings to develop practical implementation plans. These factors indicate both an opportunity and a need to propose relevant readiness domains and a practical approach to assessing them for use in primary care settings (24).

In the CRCCP, published data suggest varying levels of success in bolstering screening rates across partner clinics (15,25). Differences in clinics’ readiness to select, implement, and sustain EBIs most appropriate for their settings may contribute to the uneven changes in screening rates observed (26). To address this, CRCCP recipients in the current 5-year funding cycle (2020–2025) are required to work with clinic partners to assess 6 domains of readiness identified by CDC and use findings to develop a clinic-specific implementation plan. The 6 domains are 1) baseline CRC screening rate, 2) EBIs currently implemented, 3) EBI implementation quality, 4) workflow and screening processes, 5) electronic health record capacity, and 6) clinic resources and capacity. Recipients and their partner clinics may require practical tools to assess readiness to implement EBIs and develop relevant action plans. Internal program data suggest that existing research-based readiness assessment instruments are not always practical for CRCCP clinic contexts — in part due to their length, cost, complexity, or need for research expertise to interpret — particularly in the FQHCs and other community-based clinics in which the CRCCP is implemented.

Here, we describe the development of the Field Guide for Assessing Readiness to Implement Evidence-Based Cancer Screening Interventions in Primary Care (Field Guide). We also provide a brief overview of the publicly available Field Guide (https://www.cdc.gov/cancer/crccp/field-guide.htm) and discuss its implications for practice in the CRCCP and beyond. This tool aims to fill knowledge and practice gaps by providing practical guidance to public health practitioners who work with or in primary care settings. The tool facilitates processes to collect, evaluate, interpret, and apply assessment data to tailored action plans for more effectively implementing EBIs and increasing screening rates.

## Field Guide Development and Dissemination

**Figure 1:**
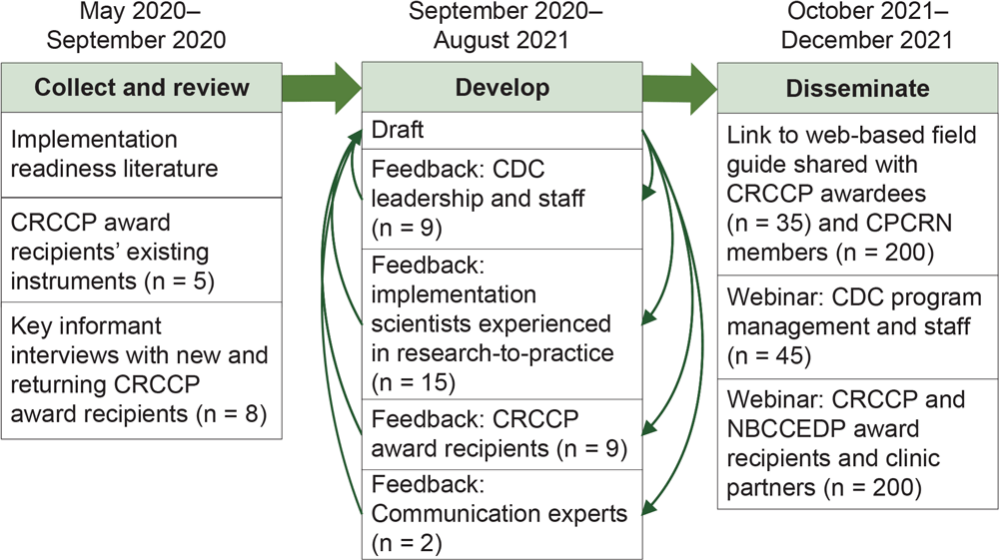
Field Guide development and dissemination for assessing readiness to implement evidence-based cancer screening interventions in primary care clinics. CDC program management and staff included staff from CDC’s Division of Cancer Prevention and Control Program Services Branch and Comprehensive Cancer Branch. Abbreviations: CDC, Centers for Disease Control and Prevention; CRCCP, Colorectal Cancer Control Program; CPCRN, Cancer Prevention and Control Research Network; NBCCEDP, National Breast and Cervical Cancer Early Detection Program.

The Field Guide development team included investigators and staff from CDC and 3 universities. Figure 1 illustrates the iterative, evidence-, and practice partner–informed process we used to develop and disseminate The Field Guide between May 2020 and December 2021.

### Identifying and characterizing relevant readiness instruments: evidence and practice review

First, we sought to identify existing instruments that recipients could use to assess a clinic’s readiness to implement EBIs and improve CRC screening. We also aimed to identify best practices for conducting readiness assessments in clinic settings and applying findings to decision making (eg, guidance on selecting which EBIs to adopt or determining gaps that clinics may need to address before implementation). We characterized potentially relevant readiness instruments and triangulated data from 1) a review of instruments described in 3 seminal systematic reviews of readiness assessment instruments in diverse health care settings (20–22), 2) a document review of CRCCP recipients’ existing practices for determining readiness, and 3) semi-structured key informant interviews with a diverse subset of CRCCP recipients. We evaluated tools on the basis of their 1) ability to meet the 6 required assessment domains, 2) length, 3) applicability to primary care settings, 4) adaptability across clinic contexts, and 5) accessibility. Applicability was assessed on the basis of an instrument’s prior use in and/or design for primary care settings. Adaptability was assessed based on an instrument’s prior use in multiple, ideally primary care, settings; multiple versions of the instruments; and inclusion of instructions for instrument modification. Accessibility was assessed on the basis of cost for users and access to scoring guides.

*Research-based instrument review.* Through our review of published readiness assessments, we determined that the more than 50 measures described primarily focused on evaluating the skills and capacity of individuals who would deliver interventions and increase organizational capacity. However, most measures did not explicitly evaluate resources available as part of assessing capacity, a concept important in resource-limited settings, particularly FQHCs involved in the CRCCP. Among all instruments described, we identified 3 with applicable measures as defined by our evaluation criteria above: the Organizational Readiness for Change (ORC) (27), Organizational Readiness for Implementing Change (ORIC) (28), and Organizational Readiness for Change Assessment (ORCA) (29). All 3 were guided by implementation theories and measured between 2 and 4 of the 6 CDC-required assessment domains. All 3 included multiple versions, and/or had been adapted and tested in multiple healthcare settings. None addressed cancer screening or were implemented in primary care settings.

*CRCCP recipient document review*. Under previous CRCCP funding cycles, some recipients had developed materials to assess domains of readiness among their partner clinics. With permission from recipients, CDC team members shared these materials with the Field Guide development team for review and content analysis. All the materials initially reviewed were quantitative and addressed most of the required assessment domains. Most were long and, although none indicated an amount of time for completion, appeared to require substantial time and effort for recipients and clinic staff to utilize. In addition, whether the materials were intended to be administered at the health system or clinic level was unclear, and they largely omitted guidance to apply assessment findings to implementation plans.

*Key informant interviews.* We followed the document review with telephone interviews with key informants. We invited 6 recipients who were continuously funded from 2015–2020 and 2020–2025. These recipients were known to have readiness assessment materials in place or had clinics that terminated before the end of the award cycle. We anticipated that this mix of recipients could provide insight into promising readiness assessment processes as well as how results of a readiness assessment could have prevented clinic termination. In addition, we invited 3 recipients newly funded for 2020–2025 to elicit perspectives of those for whom the readiness assessment requirements were new. We aimed to gain recipients’ insights about 1) the process and content of any previously conducted readiness assessments, 2) which readiness tools may be most useful, and 3) gaps where focused guidance for conducting readiness assessments may be needed. Six returning and 2 new recipients agreed to participate in an interview. The University of Washington institutional review board designated this work as exempt.

Although assessment materials provided by award recipients for review did not include qualitative instruments, interviews indicated that these did in fact exist throughout recipient programs and were an important supplement to quantitative assessment efforts. Key informants who represented current and previous recipients generally said that mixed-methods assessments conducted before implementation prepared them to use results to inform development of tailored action plans for EBI implementation and technical assistance with partners. These recipients discussed the value of meeting regularly with clinic staff during the assessment process, with greater frequency in the pre-implementation and early phases of implementation. One recipient described using a theoretical framework to inform their quantitative assessment, and others indicated interest in applying implementation theory–guided instruments if the tool made sense for the context and was practice-oriented (rather than research-focused). Some recipients cautioned that clinics scored uniformly high on quantitative assessments, whereas qualitative assessment revealed nuance in resources and contexts, a reflection further supported by the 5 recipients who shared qualitative approaches as part of their assessment materials. One recipient described conducting clinic observations to assess workflow practices and gaps regarding identification and notification of patients due for screening, follow-up with patients, and communication with endoscopy clinics, when needed. Both new and returning recipients said they would benefit from examples or templates of readiness assessments that met all CDC-required assessment domains, some ideas for best practices around engaging clinic partners, and reminders about where overlap exists in routine reporting for the CRCCP and CDC-required assessment requirements.

### Developing the Field Guide: partner feedback

We synthesized information from the evidence and practice review to draft the Field Guide, a toolkit that offers practical resources for primary care clinics and partners to assess readiness to implement EBIs. To refine the Field Guide, between September 2020 and August 2021, we sought feedback on successive drafts among the development team and multiple partner groups, including research partners, CDC leadership and staff, and CRCCP recipients (Figure 1). We expected the Field Guide to, at a minimum, enable all partners to assess CDC-required assessment domains. Given recipient and partner clinics’ varying experience implementing EBIs, we also recognized the need for partners to be able to adapt the Field Guide to meet their specific needs. For example, clinics new to the screening program may prefer to focus on the minimum required domains, while those with experience implementing EBIs as part of the CRCCP may be ready to assess additional domains. We discussed several drafts within the development team and facilitated an interactive seminar with research partners experienced in toolkit development, dissemination, and adoption. Then we sought feedback of Field Guide content among CDC leadership and program consultants who offer technical assistance to recipients. Program consultants help to ensure successful program implementation by providing technical assistance to recipients on goal setting, strategy, implementation, evaluation, and resource management. Program consultants also help balance CDC requirements and expectations with recipient program needs, experiences, and contexts. We obtained extensive feedback from a subset of CRCCP recipients who represented end users of the Field Guide and incorporated feedback after each stage of review. We anticipated eliciting feedback at this stage of development from clinic partners in addition to those described in Figure 1. However, the development phases aligned with multiple waves of COVID-19 infection, during which time primary care clinics were facing extreme challenges, including revenue and staffing reductions (30), which affected their ability to undertake additional activities. Upon conferring with CDC program consultants and award recipients who were familiar with their primary clinic partners’ capacity, we opted to focus our review on meeting the needs of the end-users of the Field Guide, in this case, award recipients.

The major changes to the Field Guide resulted from 3 primary concerns identified during the iterative review process. First, reviewers said the document was too complex, and while the phased approach was helpful, each phase contained too much information that made it difficult for recipients to decipher what was useful for them. In response, we created a checklist of activities for each phase available at the beginning of the document. This feedback further indicated that a dynamic interface would best serve the needs of recipients and clinic partners, the tool’s end users. We determined that a web-based interface, rather than the originally planned interactive .pdf format, would enable users to navigate to pertinent sections of the Field Guide for more detail when needed and skip areas that were not relevant. Second, reviewers representing CDC, program consultants, and recipients commented that the document may be too research-oriented and academic to be accessible for some practice partners. A goal of this work was to integrate implementation science and practice, so the tools we suggested needed to be practical for real-world settings. To address this challenge, we replaced references to ORC, ORIC, and ORCA with the Clinical Sustainability Assessment Tool (31) and the R = MC^2^ readiness building framework (17). These instruments both measure CDC-required domains and are tools with which some recipients were familiar. We consulted communications experts to clarify and simplify language and remove jargon. We also added direct quotes from recipients throughout the document to highlight recipient insights and experiences using their own words. Third, partner reviewers indicated that the term “toolkit” implied a static document and suggested we rebrand to include “field guide” in the final resource name.

### Finalizing and disseminating the Field Guide

**Figure 2:**
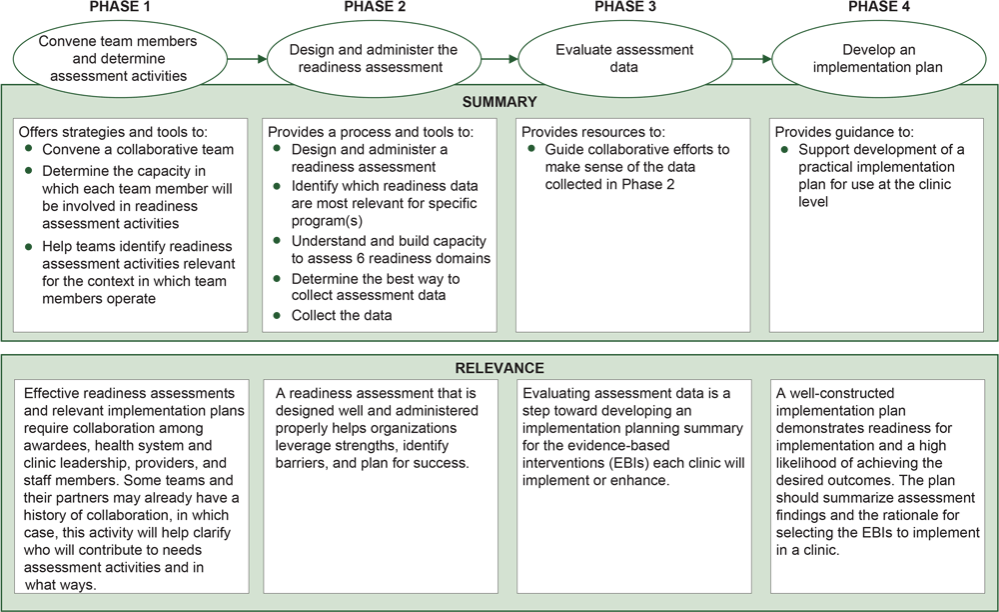
Phases of the Field Guide for assessing readiness to implement evidence-based cancer screening interventions in primary care clinics.

The Field Guide is currently hosted on the University of Washington Health Promotion Research Center website, linked via a landing page on the CDC website (https://www.cdc.gov/cancer/crccp/field-guide.htm). This publicly available web-based tool includes 4 phases (Figure 2):

1. Convene team members and determine assessment activities
2. Design and administer the readiness assessment
3. Evaluate assessment data
4. Develop an implementation plan

The Field Guide landing page provides an overview of the tool and features a downloadable activities checklist that corresponds to each phase. Table 2 depicts the high-level activities included in the checklist. The landing page links to additional web pages for each readiness assessment phase, in which activities outlined in the checklist are described in more detail. Each phase includes linked and downloadable resources to help recipients and clinic partners assess the 6 CDC-required readiness assessment domains.

**Table 2:** Field Guide for Assessing Readiness to Implement Evidence-Based Cancer Screening Interventions in Primary Care Clinics: Phases 1–4 Condensed Activities Checklist^a^

Phase number	Activity type
**Phase 1**	**Convene team members and determine readiness assessment activities**
1.1	Adapt phase 1–4 activities checklists for your setting
1.2	Establish your team
1.3	Convene your team
**Phase 2**	**Design and administer the readiness assessment**
2.1	Determine the relevance and feasibility of EBIs for the clinic setting
2.2	Understand the 6 readiness assessment domains required by the CRCCP
2.3	Review suggested readiness data collection tools
2.4	Convene team to determine data collection strategies
2.5	Gather data to assess the minimum required data elements for the readiness assessment
2.6	Review the data
2.7	Resolve any discrepancies
**Phase 3**	**Evaluate assessment data**
3.1	Calculate screening rate (see CDC’s Guide to Measuring Breast, Cervical, and Colorectal Cancer Screening Rates)
3.2	Evaluate workflow and identify gaps for each screening test
3.3	Examine how each EBI is being implemented
3.4	Determine if EBI implementation aligns with CDC’s EPG processes (note: EPG resource does not include small media and patient navigation)
3.5	Identify IT challenges that impact workflows and data reporting
3.6	Summarize assessment results in writing
**Phase 4**	**Develop implementation plan**
4.1	Share and collaboratively interpret assessment findings
4.2	Agree on gaps and efforts needed to improve screening rate reporting and validation
4.3	Identify and document gaps and resources available to implement or enhance EBIs
4.4	Agree on a minimum of 2 EBIs that should be implemented at this time; if EBIs are already implemented, identify which are appropriate for enhancement; EBIs identified for implementation or enhancement should:
4.5	Document processes, team members involved, and leadership support for EBI implementation and/or enhancement
4.6	Develop a plan to address IT challenges, including IT processes to:
4.7	Plan for long-term sustainability

Abbreviations: CDC, Centers for Disease Control and Prevention; CRCCP, Colorectal Cancer Control Program; EBIs, evidence-based interventions; EPG, EBI Planning Guide; IT, information technology.

a The complete Activities Checklist, which includes additional substeps and activities, is available at https://www.cdc.gov/cancer/crccp/field-guide.htm.

In October 2021, The Field Guide was disseminated via a web link across all CRCCP (n = 35) recipients, as well as the 200-member Cancer Prevention and Control Research Network, comprising researchers, practice partners, and scholars (Figure 1). In November and December 2021, the Field Guide development team introduced the guide via webinars with CDC’s Division of Cancer Prevention and Control Program Services and Comprehensive Cancer Control Branches and a National Association of Chronic Disease Directors–hosted webinar for CRCCP and National Breast and Cervical Cancer Early Detection Program (NBCCEDP) award recipients and staff.

## The Future of the Field Guide: CRCCP and Beyond

A total of 35 CRCCP recipients representing diverse organizations including state health departments, universities, and tribal organizations received funding in 2020 to work with primary care clinics. Given the extensive reach of the CRCCP, the Field Guide has potential for wide use by award recipients to both update existing readiness assessment tools and conduct the required assessments with new clinic partners as the funding period progresses. Although the Field Guide was initially designed for CRCCP recipients and their clinic partners, it is readily applicable to CDC’s NBCCEDP, which currently funds 70 recipients that include all 50 states, the District of Columbia, 6 US territories, and 13 tribes or tribal organizations. The NBCCEDP, which is preparing for a new 5-year funding cycle to begin in 2022, provides breast and cervical cancer screening to patients with lower income and those who are under- and uninsured across the country. Like the CRCCP, one of the primary foci of the NBCCEDP is supporting partnerships with primary care clinics, promoting health equity, and improving cancer screening through EBI implementation (32). NBCCEDP recipients, some of whom are also CRCCP recipients, are likewise required to assess clinic readiness for implementation, suggesting the Field Guide’s applicability in that program. In addition to cancer-focused programs, the Field Guide may be tailored to assess clinic-level readiness to implement EBIs for other chronic diseases, as the concepts it addresses, such as convening teams, assessing capacity, selecting appropriate EBIs to fit clinic needs and resources, and translating findings into implementation plans, are relevant to implementing any new evidence-based practice, policy, or program.

Despite its strengths and potential for use across multiple settings, the Field Guide has limitations. Our development process did not include clinic-level representatives, given the extraordinary burden clinics were facing because of the COVID-19 pandemic. Although some recipients were primary care providers, they represented recipients and not clinic partners, whose perspective could strengthen the tool’s utility. We engaged as many diverse partners as possible in Field Guide development, but it may omit factors that are important to some partners whose perspectives were not reflected here. Additionally, the example instruments included in the guide have not been validated in primary care settings. A formal pilot test to evaluate use of the tool in practice across a wider sample of partners could address these limitations. Our team plans to evaluate use of the Field Guide in practice as an important next step to assess validity and reliability and to advance practical implementation science — that is, integrating implementation theory into resources that are relevant and applicable in real-world primary care settings (33).

The Field Guide integrates implementation science theory and practical experience into a relevant tool to help assess and bolster clinic capacity to implement cancer screening EBIs, and it addresses a current practice gap among primary care clinics and their partners who are working to achieve greater increases in CRC screening. Furthermore, the Field Guide is a promising tool for reducing cancer disparities, given its focus on making implementation science tools relevant for FQHC settings, where underserved patients with low screening rates and a high burden of CRC, limited access to CRC screening, and suboptimal CRC screening rates receive care.
